# Increased Risk of End-Stage Renal Disease (ESRD) Requiring Chronic Dialysis is Associated With Use of Nonsteroidal Anti-Inflammatory Drugs (NSAIDs): Nationwide Case-Crossover Study

**DOI:** 10.1097/MD.0000000000001362

**Published:** 2015-09-25

**Authors:** Yu-Kang Chang, Jia-Sin Liu, Yueh-Han Hsu, Der-Cherng Tarng, Chih-Cheng Hsu

**Affiliations:** From the Institute of Population Health Sciences, National Health Research Institutes, Zhunan (Y-KC, J-SL, C-CH); Department of Health Services Administration, China Medical University, Taichung (C-CH); Division of Nephrology, Department of Internal Medicine, Ditmanson Medical Foundation Chia-Yi Christian Hospital, Chiayi (Y-HH); Department of Nursing, Min-Hwei Junior College of Health Care Management, Tainan (Y-HH); Institutes of Physiology and Clinical Medicine, National Yang-Ming University (D-CT); Division of Nephrology, Department of Medicine, Taipei Veterans General Hospital (D-CT); and Institute of Clinical Medicine, National Yang-Ming University, Taipei, Taiwan (D-CT, C-CH).

## Abstract

It is known that many medical adverse events can be caused by nonsteroidal anti-inflammatory drugs (NSAIDs); however, epidemiologic evidence has not granted an affirmative relationship between NSAID use and the risk of end-stage renal disease (ESRD). We aimed to investigate the relationship in a Chinese population between short-term NSAID use and development of ESRD requiring chronic dialysis.

A retrospective case-crossover design was used in this study. Using the Taiwanese National Health Insurance database, we identified 109,400 incident chronic ESRD patients with dialysis initiation from 1998 to 2009. For each patient, we defined the case period as 1 to 14 days and the control period as 105 to 118 days, respectively, before the first dialysis date. The washout period was 90 days between the case and control period. Detailed information about NSAID use was compared between the case and control periods. We calculated odds ratios (ORs) and their 95% confidence intervals (CIs) using a conditional logistic regression model.

NSAID use was found to be a significant risk factor associated with dialysis commencement. The adjusted OR was 2.73 (95% CI: 2.62–2.84) for nonselective NSAIDs and 2.17 (95% CI: 1.83–2.57) for celecoxib. The OR reached 3.05 for the use of acetic acid derivatives. Compared with the oral forms, significantly higher risks were seen in parenteral NSAID use (OR: 8.66, 95% CI: 6.12–20.19).

NSAIDs should be prescribed with caution, especially for those in ESRD high-risk groups.

## Abstract 2

Supplemental Digital Content is available in the text

## INTRODUCTION

Nonsteroidal anti-inflammatory drugs (NSAIDs) are the most common medications used by the general population for acute or chronic soreness and inflammation. It has been estimated that >30 million people worldwide take NSAIDs every day.^1^ The systemic adverse consequences caused by NSAIDs—such as acute myocardial infarction,^2–4^ stroke,^5^ gastrointestinal bleeding,^6–8^ and renal cancer^9^—are well known. Regarding the NSAID-related renal effects, some harmful pathologic dynamics have been recognized, which include sodium and water retention,^10,11^ hyperkalemia,^12^ acute renal injury,^13^ interstitial nephritis,^14^ nephrotic syndrome,^15^ papillary necrosis,^16^ and developmental defects^17^; however, the relationship between NSAID use and development of end-stage renal disease (ESRD) requiring dialysis has not been well established in epidemiologic studies. In a case–control study, Perneger et al^18^ showed a cumulative dose of >5000 pills containing NSAIDs was associated with an increased risk of long-term dialysis. Schneider et al^19^ found that both selective and nonselective NSAIDs had a significant association with acute renal failure in a nested case–control study; however, many more studies have reported conflicting results. Murray et al^20^ indicated regular analgesic consumption, including the use of NSAIDs, did not increase the risk of ESRD development. Ibanez et al^21^ could not link the risk of ESRD to long-term use of NSAIDs. In a recent study, Michielsen et al^22^ also could not affirm the association between analgesic nephropathy and high use of nonphenacetin analgesics in ESRD patients. The inconsistent results may derive from some inherent limitations for the case–control studies: the researchers could not completely guarantee comparability between the case and control groups; the recall bias pertaining to lifelong NSAID use in interview surveys could also significantly jeopardize accurate estimate of renal impacts caused by NSAID use. In this study, we used a case-crossover study design to eliminate any undetectable residual confounding factors to delineate the risk of NSAID use in the development of ESRD requiring chronic dialysis. Furthermore, we used a reliable National Health Insurance (NHI) dataset^23^ to precisely calculate NSAID dosage and define the time at which dialysis commenced. We aimed to estimate the unbiased risk encountered by NSAID users for the initiation of dialysis.

## MATERIALS AND METHODS

### Data Source

The NHI program, which enrolls about 99% of Taiwan's population of 23 million, has been the country's core infrastructure of the health care system since 1995. The Taiwan National Health Insurance Research Database (TNHIRD), derived from the reimbursement claims within the NHI program, is considered one of most reliable national medical utilization databases in the world.^23^ From the TNHIRD, we retrieved as our study subjects a cohort who received at least 3-month dialysis treatment (chronic dialysis) during 1998 to 2009. To search for comorbidities that the study subjects might have, we also studied the TNHIRD with antecedent data from January 1, 1997. This study has been approved by the institutional review board of the National Health Research Institutes in Taiwan.

### Design and Study Subjects

We used a population-based, case-crossover study design to evaluate the relationship between short-term NSAID use and the risk of developing ESRD requiring chronic dialysis.^24^ The subject-as-own-control research design, in which the frequency of NSAID exposure in the case and control periods were retrospectively compared, has advantages in reducing recall bias, selection bias, and undetectable residual confounding factors because the exposure information in the case and control periods is provided by the same person. If the exposure time is short (2 weeks in the current study), bias from misclassification and time-varying exposure status can further be reduced.^25^ We identified all patients who started chronic dialysis from 1998 to 2009 based on the dialysis treatment codes of TNHIRD. The patients under hemodialysis or peritoneal dialysis were all selected. The date of the first dialysis was defined as the index date. There were 109,400 subjects selected in the current study.

### Data on Drug Exposure

The drug exposures of interest in this study were collected from the reimbursement claims recorded in the TNHIRD. We divided the nonselective NASIDs into 5 classifications, based on their mechanism of action or chemical structure: aspirin, propionic acid, acetic acid derivatives, enolic acid derivatives, and anthranilic acid derivatives. Several commonly used NSAIDs were then classified accordingly: the propionic acid category included ibuprofen, naproxen, ketoprofen, fenbufen, alminoprofen, and flurbiprofen; the acetic acid derivatives included indomethacin, alclofenac, diclofenac, etodolac, ketorolac, and sulindac; the enolic acid derivatives included meloxicam, piroxicam, tenoxicam, and nimesulide; and the anthranilic acid derivatives included flufenamic acid, mefenamic acid, niflumic acid, tiaprofenic acid, and tolfenamic acid. For the selective NSAIDs, we included only 2 drugs used in the Taiwan market: celecoxib and rofecoxib. From the pharmacy dispensing records of the TNHIRD, we collected information about the type of drugs, dosage, date of prescription, total number of drug pills, and days of drug supply. We used the WHO standard methods^26^ to calculate the defined daily dose of NSAIDs taken by each study subject to assess their drug exposure in the case and control periods. Then we further stratified those who had taken NSAIDs according to quartile of defined daily dose per day and different cumulative exposure time (1–3, 4–6, 7–10, or 11–14 days) for subgroup analyses.

### Comorbidies and Concomitant Medications

We collected patients’ information on sex, age, date of dialysis commencement, and comorbidities from the TNHIRD. The following chronic comorbidities were defined as covariates, if the subjects, prior to dialysis commencement, had at least 1 hospitalization or 2 ambulatory visits within 1 year due to any of the following illnesses: diabetes mellitus (International Classification of Diseases, 9th Revision, Clinical Modification [ICD-9-CM] code 250), hypertension (ICD-9-CM codes 401–405), cardiovascular disease (ICD-9-CM code 410–414), gout (ICD-9-CM codes 274.9), cancer (ICD-9-CM codes 140–208), stroke (ICD-9-CM codes 430–438), and myocardial infarction (ICD-9-CM codes 410). Two acute illnesses—urinary tract infection (ICD-9-CM codes 599.0) and pneumonia (ICD-9-CM codes 486)—were identified in the case period. Other concomitant drug use collected in this study included acetaminophen, antihypertensive agents (angiotensin-converting enzyme inhibitors, angiotensin receptor blockers, beta-blocker, calcium channel blockers, and diuretics), antidiabetic drugs (biguanides, sulfonylureas, and insulin), and statins. We further collected information on contrast media and aminoglycosides use in the case period to minimize confounding effects caused by these drugs.

## STATISTICAL ANALYSIS

For each study subject, the case period was defined as 1 to 14 days before the index date and the control period as 105 to 118 days before the index date, by which we set 90 days between the case and control periods as a washout period to minimize drugs’ residual effects. The cumulative dosages of NSAID prescriptions during the case and control periods were respectively calculated. We compared the odds of NSAID use between the case and control periods to estimate the crude odds ratio (OR) and 95% confidence interval (CI). In the multiple analyses, conditional logistic regressions were performed to assess excess risk of starting dialysis for NSAID use after adjusting for sex, age, comorbidity, year of dialysis commencement, and concomitant medications used. To evaluate the possible dose–response effect, we also separately estimate the adjusted OR for those with different exposure dosage (by quartile) and different exposure duration (1–3, 4–6, 7–10, or 11–14 days). In the sensitivity analyses, we repeated our conditional logistic analyses by stratifying study subjects according to their sex (male, female), age (<40, 40–64, ≥65), year of dialysis commencement (1998–2003, 2004–2009), comorbidities including diabetes, hypertension, gout, cardiovascular disease, cancer, stroke, and myocardial infarction, and some acute events—including intensive care unit (ICU) admission, contrast media use, urinary tract infection, and pneumonia—during the case period. We also evaluated the robustness of our results by defining different washout period (60, 90, 120, and 180 days).

All p values were 2-sided, with a *P* value < 0.05 considered to have reached a significance level. All analyses were conducted using SAS version 9.2 (SAS Institute Inc, Cary, NC).

## RESULTS

We identified 100,490 patients with dialysis commencement from 1998 to 2009. The baseline characteristics of the study subjects are summarized in Table 1. Approximately half of the selected patients were male and >65 years. Diabetes (50.1%) and hypertension (65.2%) were the 2 main comorbidities the study subjects had prior to development of dialysis, followed by cardiovascular disease (32.7%) and myocardial infarction (23.9%). Table 2 presents the use of concomitant medications during the case period (1–14 days before the index date) and the control period (105–118 days before the index date). A similar pattern of drug prescription for diabetes and hypertension could be seen in the case and control periods. Nevertheless, the use of contrast media during the case period was significantly more frequent than in the control period (13.1% vs 1.8%). Disproportional distribution was also found for aminoglycosides use (5.6% vs 3.5%).

**TABLE 1:**
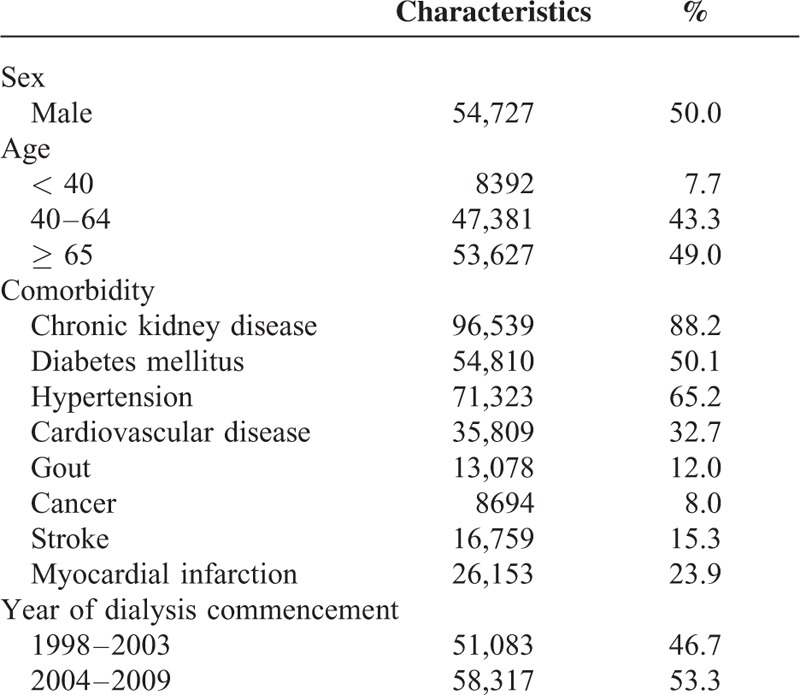
Baseline Characteristics of the Study Subjects (n = 109,400)

**TABLE 2:**
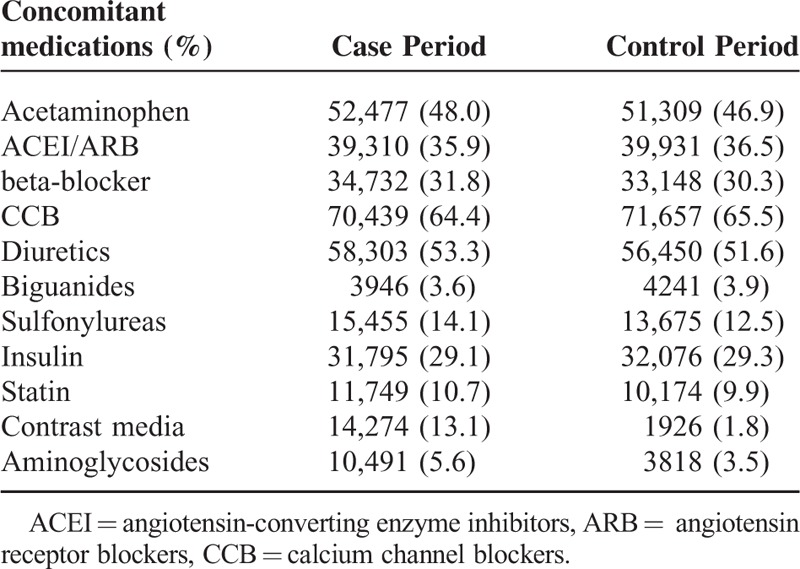
Prescriptions of Concomitant Medications during Days 1–14 (Case Period) and Days 105–118 (Control Period) before Initial Dialysis (n = 109,400)

Table 3 shows the crude and adjusted ORs for the development of ESRD requiring chronic dialysis under the use of NSAIDs. The adjusted ORs of overall, selective, and nonselective NSAID use were 2.72 (95% CI: 2.60–2.82), 1.70 (95% CI: 1.51–1.91), and 2.73 (95% CI: 2.62–2.84), respectively. For individual classes of nonselective NSAIDs, the highest adjusted OR was 3.05 (95% CI: 2.87–3.23) for acetic acid derivatives use; the risks of use of enolic acid derivatives, propionic acid, anthranilic acid derivatives, and aspirin were 1.91 (95% CI: 1.74–2.11), 1.68 (95% CI: 1.55–1.82), 1.65 (95% CI: 1.49–1.79), and 1.53 (95% CI: 1.41–1.69), respectively. The adjusted ORs of selective NSAIDs for celecoxib and rofecoxib were 2.17 (95% CI: 1.83–2.57) and 1.65 (95% CI: 1.23–3.35), respectively. The highest adjusted OR found the parenteral NSAID use (OR: 8.66, 95% CI: 6.12–20.19).

**TABLE 3:**
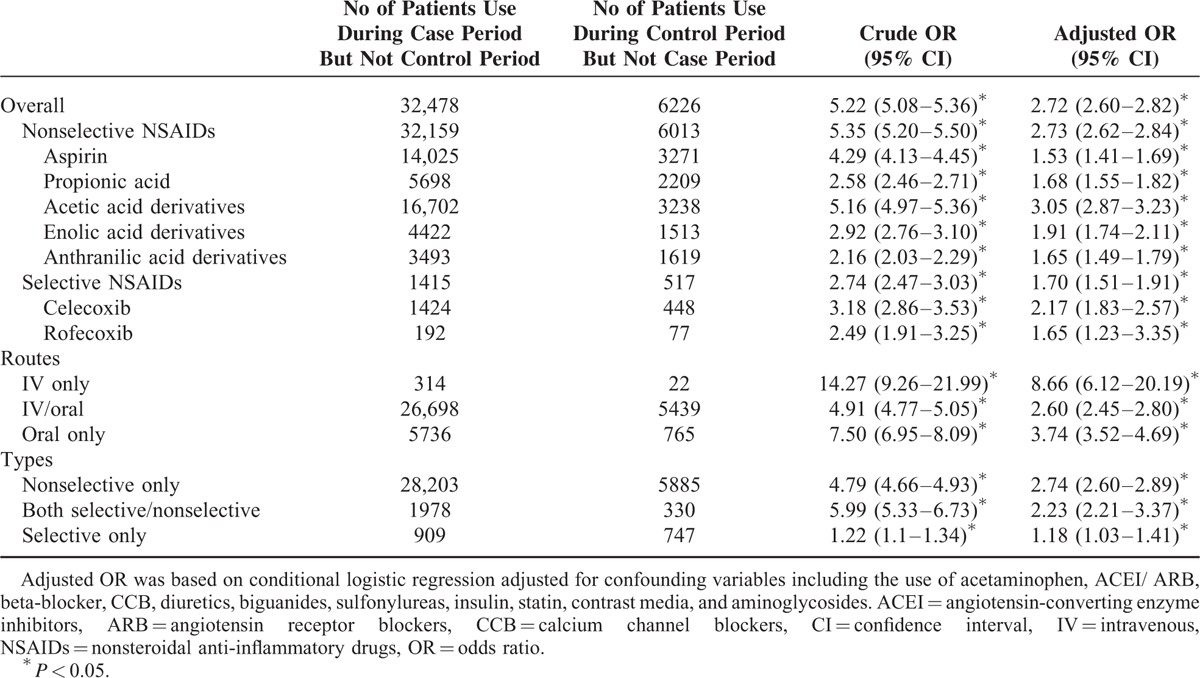
Risk of Chronic Dialysis Associated With Current Use of Selective and Nonselective NSAIDs

**FIGURE 1:**
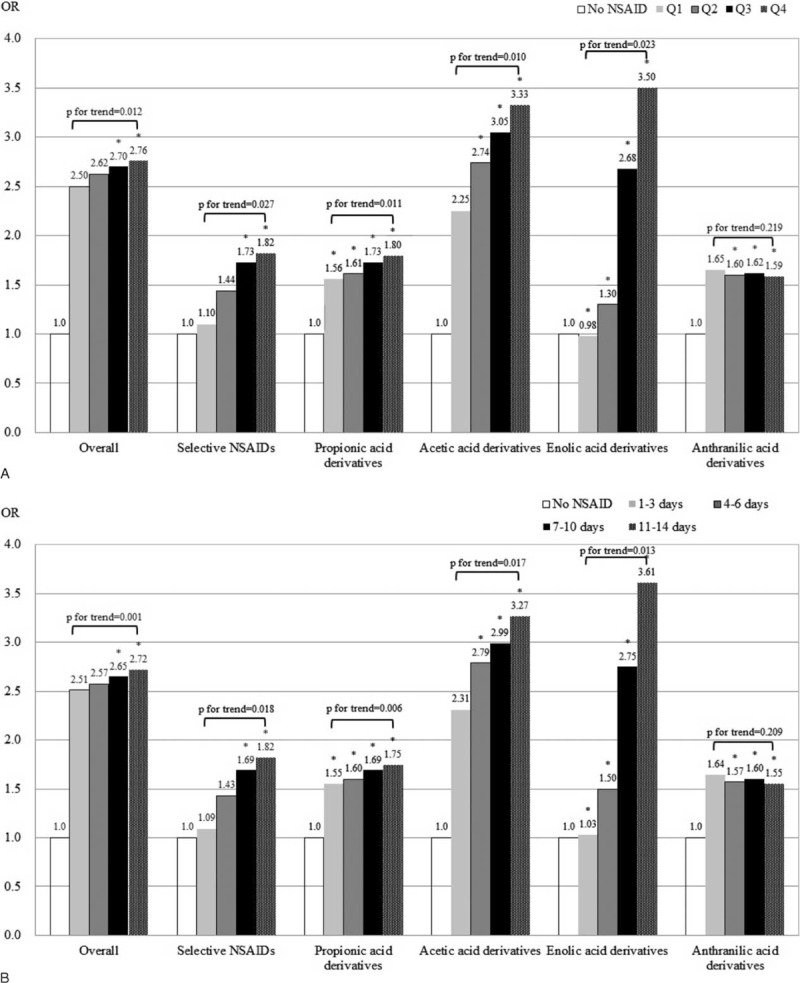
Risks of end-stage renal disease requiring chronic dialysis associated with short-term exposure of nonsteroidal anti-inflammatory drugs, stratified by different exposure dosage A, and B, duration. (Q1: ≤0.25, Q2: 0.26–0.50, Q3:0.51–0.75, Q4: >0.75 define daily dose, ^∗^*P* < 0.05). NSAIDs = nonsteroidal anti-inflammatory drugs, OR = odds ratio.

Figure 1 shows dose–response renal risks in the overall, selective, and different nonselective NSAID classes. Compared with the nonusers, the renal risk was gradually increased with higher NSAID cumulative exposure dosage (Panel A) and days (Panel B) (except the anthranilic acid derivatives, *P* for trend were all significant in different NSAID drug classes). The detailed information about dose–response effects were shown in supplemental Table 1, http://links.lww.com/MD/A417 and supplemental Table 2, http://links.lww.com/MD/A417. The estimated effects of NSAID use on development of chronic dialysis were similar to the main findings, irrespective of whether the washout period was redefined (supplemental Table 3, http://links.lww.com/MD/A417).

**FIGURE 2:**
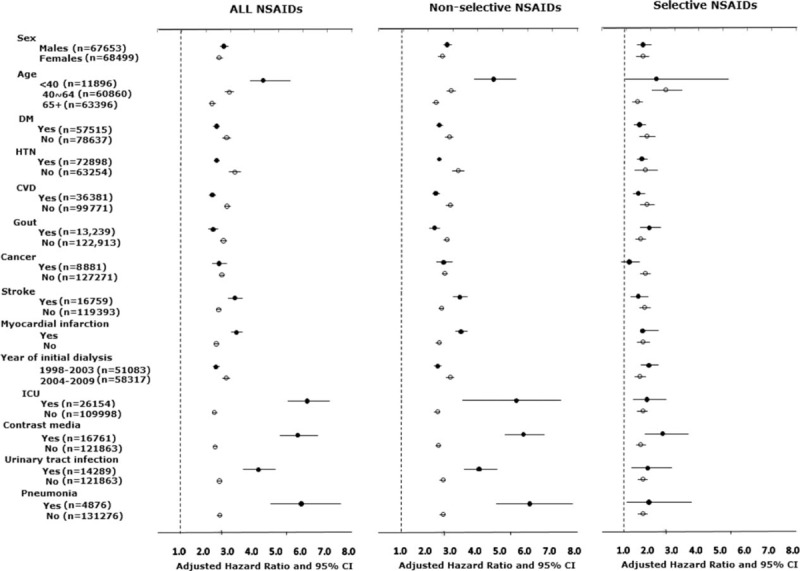
Risk of chronic dialysis associated with current use of nonsteroidal anti-inflammatory drugs, stratified by sex, age, comorbidity status, year of dialysis commencement, and acute medical events that occurred during the case period. CVD = cardiovascular disease, DM = diabetes mellitus, HTN = hypertension, ICU = intensive care unit, NSAIDs = nonsteroidal anti-inflammatory drugs, OR = odds ratio.

In sensitivity analyses (Figure 2), NSAIDs, both selective and nonselective, were associated with a higher risk of developing ESRD requiring dialysis; this was consistent across different patient characteristics, comorbidities, and acute events in the case period. Generally speaking, the risk of NSAID-related initiation of dialysis was higher in men, younger people, and those without chronic diseases such as diabetes, hypertension, chronic kidney disease, and gout. In contrast, those who suffered from acute illness in the case period—such as ICU hospitalization, contrast media use, urinary tract infection, and pneumonia—were more sensitive to the harmful effects of NSAID use. Higher dialysis risks were noted for concurrent use of NSAIDs during the acute illness stage.

## DISCUSSION

This is the first study investigating the risk of development of ESRD requiring chronic dialysis for short-term exposure to NSAIDs. The current study demonstrated that most selective or nonselective NSAIDs were associated with higher risk of dialysis commencement. The use of parenteral forms of NSAIDs could even increase the dialysis risk by up to 8 times. We used a case-crossover study design to resolve common limitations encountered in traditional case–control studies.^27,28^ The information on NSAID exposure in this study was retrieved from the reliable TNHIRD, so we could eliminate recall bias. Different from other studies^18,21^ in which lifelong exposures were often inquired, this study could accurately assess impacts of immediate NSAID use on renal function deterioration. Our results clearly show that NSAID exposure could be the last straw triggering the necessities for dialysis commencement. Clinicians should be cautious about prescribing any NSAIDs to vulnerable ESRD patients.

In a nested case–control study, Schneider et al^19^ found that the elderly in the Canadian Healthcare Program who were the current NSAID users were more likely to be hospitalized for acute renal failure; however, the effects of renal risk would be diminished after ≥30 days without NSAID prescription. Huerta et al^29^ used the British General Practice Research Database to conduct another nested case–control study, resulting in similar conclusions: compared with nonusers, current NSAID users increased their risk of acute renal failure by 3.2-fold; but this hazard ratio declined and became insignificant once NSAID use had been stopped for >30 days. Apart from being consistent with these previous findings, the current study is the first study to further demonstrate that short-term NSAID use could cause not only transient acute renal failure but also permanent kidney damage requiring dialysis, a final detrimental renal outcome.

We found that selective and nonselective NSAID users alike were both prone to develop ESRD requiring chronic dialysis; but the risk of nonselective NSAIDs (adjusted OR: 2.73) were generally higher than selective NSAIDs (adjusted OR: 1.70). We also reported a higher adjusted OR for celecoxib (2.17) than for rofecoxib (1.65), which was in accord with some research but different from others.^26,30–34^ The discrepant renal impacts between celecoxib and rofecoxib illustrated in previous research may be due to differences in study designs, sample sizes, observation times, and definitions of NSAID exposure and renal outcomes. Of the nonselective NSAIDs, almost all the derived classifications were shown to be potentially harmful to renal function; the worst were the acetic acid derivatives (adjusted OR: 3.05), such as indomethacin, sulindac, ketorolac, and diclofenac, which are all commonly used analgesics in health care facilities.

The highest risk identified in the current study to cause dialysis commencement was the use of the parenteral form of NSAIDs. Compared with those who did not take NSAID, users of oral NSAID were 3.74 times more likely to develop ESRD requiring chronic dialysis; but this severe renal risk could be even greater for the people who had used the parenteral form of NSAIDs within 2 weeks (adjusted OR: 8.66). There have been few studies investigating kidney dysfunction induced by parenteral NSAID use. Previously, Feldman et al^35^ compared use of parenteral opioid analgesics and use of parenteral ketorolac, and found the latter would increase the risk of acute renal failure (adjusted OR: 2.08) only when parenteral ketorolac was used for >5 days. More recently, parenteral NSAID users were found to be more likely to have acute myocardial infarction^4^ and stroke^5^ strengthening concerns about the detrimental renal impact of parenteral NSAIDs. Furthermore, in spite of the theoretically equal efficacy between the oral and parenteral forms of various NSAIDs,^36–38^ both patients and physicians still believed parenteral forms were more potent than the oral forms.^39,40^ Thus, prescription of parenteral NSAIDs is common among general practitioners and in emergency departments. Our study provides empirical evidence for greater precaution in prescribing parenteral NSAIDs.

The results shown in the current study also indicate that the renal risk caused by short-term NSAID use was more likely to occur when patients were vulnerable, such as being admitted to ICU (adjusted OR: 6.17), receiving contrast medium for diagnosis/intervention procedures (adjusted OR: 5.89), having a urinary tract infection (adjusted OR: 4.09), or having pneumonia (adjusted OR: 5.86). The detrimental renal damage might have been preventable if NSAIDs had not been concurrently used.

## STUDY LIMITATIONS

The results presented in the current study should be interpreted with caution due to the following limitations. First, in examining drug exposure we did not account for over-the-counter NSAID use; but the nondifferential misclassification of exposure status would probably bias the results toward the null hypothesis. Second, the case-crossover design is a retrospective observational study, so we cannot make causal inferences. Third, there are no behavioral or lifestyle profiles in the TNHIRD, so we could not control for confounding factors such as body mass index, cigarette smoking, alcohol consumption, and clinical biomarkers; however, these unmeasurable confounding factors would keep consistent during the relatively short time between the case and control period, minimizing the limitations inherent in research using healthcare insurance databases. Fourth, our study evaluated the risk of short-term NSAID use; the results might not be generalizable to the risk of long-term NSAID use. Fifth, due to availability of data sources, we could only assess renal risk of NSAID use for those under chronic dialysis, but we believed consistent results could be seen in patients with acute kidney injury.

## CONCLUSIONS

Use of selective COX inhibitors or nonselective NSAIDs, for even as little as 2 weeks, may be able to deteriorate vulnerable people's renal function to a level requiring dialysis. Health providers should be cautious about prescribing NSAIDs, particularly in a parenteral form, to any patient at risk of renal function impairment.
